# Computed Tomography Coronary Angiogram as a First-Line Diagnostic Tool in Pregnancy-Associated Spontaneous Coronary Artery Dissection: A Case Report of Left Main Stem and Left Anterior Descending Artery Dissection at 35 Weeks' Gestation

**DOI:** 10.7759/cureus.95615

**Published:** 2025-10-28

**Authors:** Kiran Sheikh, Sara Ali, Divya Gunesh

**Affiliations:** 1 Cardiology, William Harvey Hospital, Kent, GBR

**Keywords:** conservative management, ct coronary angiography, myocardial infarction in pregnancy, risks of invasive angiogram, spontaneous coronary artery dissection

## Abstract

Spontaneous coronary artery dissection (SCAD) is an uncommon cause of acute myocardial infarction, typically affecting younger individuals without traditional risk factors for atherosclerotic cardiovascular disease. The underlying pathophysiology differs entirely from that of atherosclerotic myocardial infarction, necessitating distinct diagnostic and management strategies. When SCAD occurs during pregnancy, it is termed pregnancy-associated SCAD (P-SCAD). Because of its rarity, no standardized guidelines currently exist for the diagnosis and management of P-SCAD.

We report the case of a 37-year-old woman at 35 weeks’ gestation, with no traditional atherosclerotic risk factors, who presented with chest pain. Her electrocardiogram (ECG) showed subtle changes suggestive of anterior ST-elevation myocardial infarction. Considering the procedural risks associated with an invasive coronary angiogram, a computed tomography (CT) coronary angiogram was performed first, confirming dissection involving the left main stem and proximal left anterior descending (LAD) artery. She experienced a brief episode of pulmonary edema that responded well to furosemide and subsequently remained hemodynamically stable. The patient was monitored in the hospital and later delivered uneventfully about four weeks after the event.

This case highlights the value of CT coronary angiogram as a non-invasive diagnostic option in hemodynamically stable antepartum patients with suspected SCAD, providing a safe alternative to invasive angiography in selected cases.

## Introduction

Spontaneous coronary artery dissection (SCAD) is angiographically classified into three main types and almost universally presents as acute coronary syndrome (ACS) [1]. It is estimated to account for 1%-4% of all ACS cases [1]. Although pregnancy-associated SCAD (P-SCAD) represents approximately 10% of all SCAD cases, it accounts for 21%-27% of ACS cases occurring during pregnancy and nearly half of postpartum acute myocardial infarction cases [2].

Current guidelines provide general recommendations for SCAD management but lack specific guidance for pregnant patients, in whom both maternal and fetal safety must be carefully balanced.

We present a case of SCAD involving the left main stem and left anterior descending (LAD) artery, diagnosed at 35 weeks’ gestation using computed tomography (CT) coronary angiogram. This non-invasive approach was chosen over conventional invasive angiography to minimize maternal and fetal risk. This case underscores the current gap in the literature regarding SCAD during pregnancy and highlights CT coronary angiogram as a potential first-line diagnostic tool in hemodynamically stable patients.

## Case presentation

**Figure 1:**
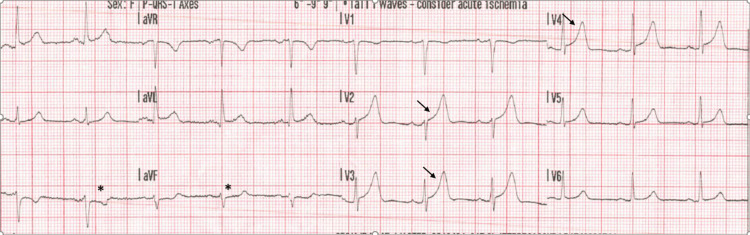
Electrocardiogram on presentation. Hyperacute T-wave in the anterior leads (arrows) and reciprocal ST depressions in lead III and AvF (asterisk).

A 37-year-old woman at 35 weeks’ gestation, gravida 2 para 0+1, developed sudden-onset chest pain in the morning while getting into the shower. The pain was central, crushing in nature, radiating to the left arm, and rated 10/10 in intensity. It was accompanied by diaphoresis, dizziness, and shortness of breath. She immediately contacted emergency services. The ambulance crew obtained an electrocardiogram (ECG), which showed hyperacute T-waves in the anterior leads and reciprocal ST depressions in the inferior leads, findings suggestive of an anterior ST-elevation myocardial infarction (Figure 1).

She was transported to the Accident & Emergency department of a local district general hospital equipped with 24/7 primary percutaneous coronary intervention (PPCI) services. Upon arrival, she experienced another episode of severe central chest pain. A lack of familiarity with SCAD among non-cardiac healthcare professionals, combined with the absence of traditional cardiovascular risk factors, resulted in a low index of suspicion for ACS and contributed to some delay in management.

She denied any history of palpitations, presyncope, or syncope, and reported no recent flu-like illness or identifiable physical or emotional stressors. She was an ex-smoker, having quit five years earlier, with no known history of diabetes, hypertension, dyslipidemia, or family history of premature atherosclerotic disease. Her obstetric history included a ruptured ectopic pregnancy treated with laparoscopic salpingectomy eight years prior. The current pregnancy was achieved through in vitro fertilization, and she had been on progesterone pessaries until 34 weeks’ gestation. 

On examination, her blood pressure was 110/70 mmHg, and heart rate was 89 beats per minute (bpm), with no radio-radial or radio-femoral delay. Chest auscultation was clear, and heart sounds were normal. The remainder of the examination was unremarkable for a 35-week gravid patient.

Initial troponin I measured one hour after symptom onset was elevated at 44 ng/L (reference range: 0-16 ng/L), rising to 27,388 ng/L at three hours. C-reactive protein was 13 mg/L (reference: 0-10 mg/L), D-dimer was 0.96 µg/mL (reference: 0.05-0.5 µg/mL), and hemoglobin was 118 g/L (reference: 110-150 g/L).

Transthoracic echocardiography demonstrated a non-dilated left ventricle (LV) with mildly impaired systolic function. The estimated ejection fraction was 45%-50%, with hypokinesia of the mid to apical anterior and septal walls. Right ventricular size and function were normal, as were biatrial dimensions and valvular structures.

Following multidisciplinary discussion involving the PPCI team, obstetricians, and the patient, it was decided to perform a CT coronary angiogram to establish the diagnosis. The scan included 3D post-processing and was performed using a systolic protocol with prospective ECG gating (heart rate: 75 bpm). She received 80 mL of Omnipaque intravenous contrast and 800 mcg of sublingual nitroglycerin. Image quality was good, with a total dose-length product (DLP) of 274 mGy·cm. 

**Figure 2:**
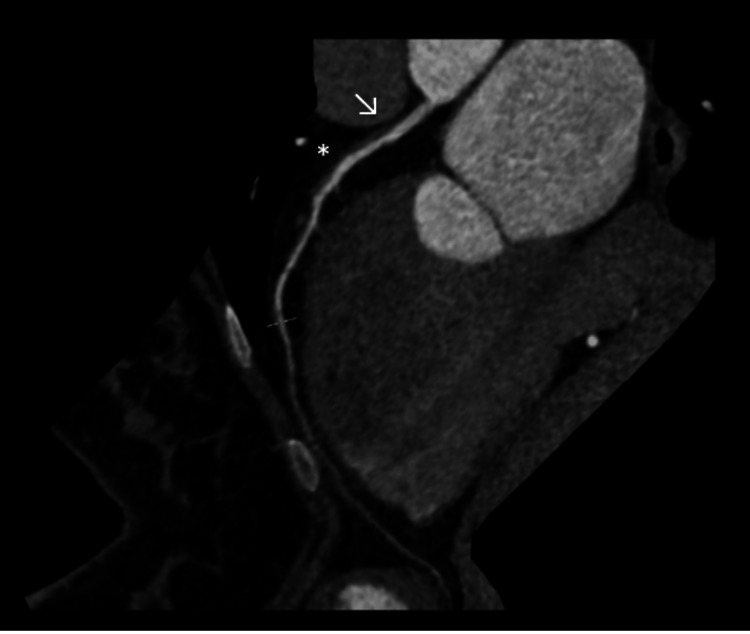
CT coronary angiogram images showing dissection involving the left main stem and proximal left anterior descending artery. An eccentric, long-segment linear hypodensity with associated wall haziness is seen in the left main stem artery (arrow), extending into the proximal LAD (asterisk).

The CT coronary angiogram revealed a dominant right coronary artery (RCA), which was unobstructed and free of plaque or stenosis. A long, eccentric linear hypodensity with associated wall haziness was noted in the left main stem artery, extending into the proximal and mid segments of the LAD artery, causing mild luminal stenosis (Figure 2). Although no definite intimal flap was visualized, the clinical context strongly suggested spontaneous coronary artery dissection, with thrombosis considered less likely. The distal LAD was unobstructed, and the first and second diagonal branches appeared normal. The left circumflex (LCx) and obtuse marginal (OM) arteries were also normal, with no evidence of plaque or stenosis. Subendocardial hypodensity was noted in the LV apex, consistent with subendocardial myocardial infarction.

She was transferred to the coronary care unit, where she was loaded with aspirin, which was then continued at a maintenance dose. An oral beta-blocker was initiated due to its role in reducing shear stress on vessel walls. ACE inhibitors were withheld, given her pregnancy. Considering the maintained flow in the dissected LAD artery and the absence of hemodynamic or electrical instability, a conservative management approach was adopted.

Later that day, she developed breathlessness without recurrence of chest pain. ECG findings remained unchanged. Chest examination revealed bilateral basal crepitations, with no additional sounds or murmurs on cardiovascular examination. Her vital signs were stable, except for oxygen desaturation on room air. She was managed for acute pulmonary edema, which resolved within an hour following intravenous furosemide and supplemental oxygen.

Due to the lack of advanced obstetric cardiac care and on-site cardiac surgery, her case was discussed with the nearest tertiary center, and she was transferred there for continued observation.

**Figure 3:**
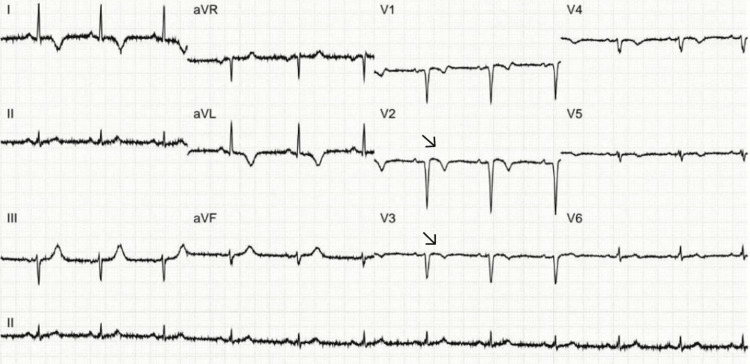
Electrocardiogram obtained a few days later showing changes of an evolved anterior myocardial infarction. Settling ST-elevations and T-wave inversions (arrows) are noted in the anterior leads.

An ECG performed a few days later showed changes consistent with an evolved myocardial infarction, with resolving ST-segment elevations and T-wave inversions in the anterior leads (Figure 3).

**Figure 4:**
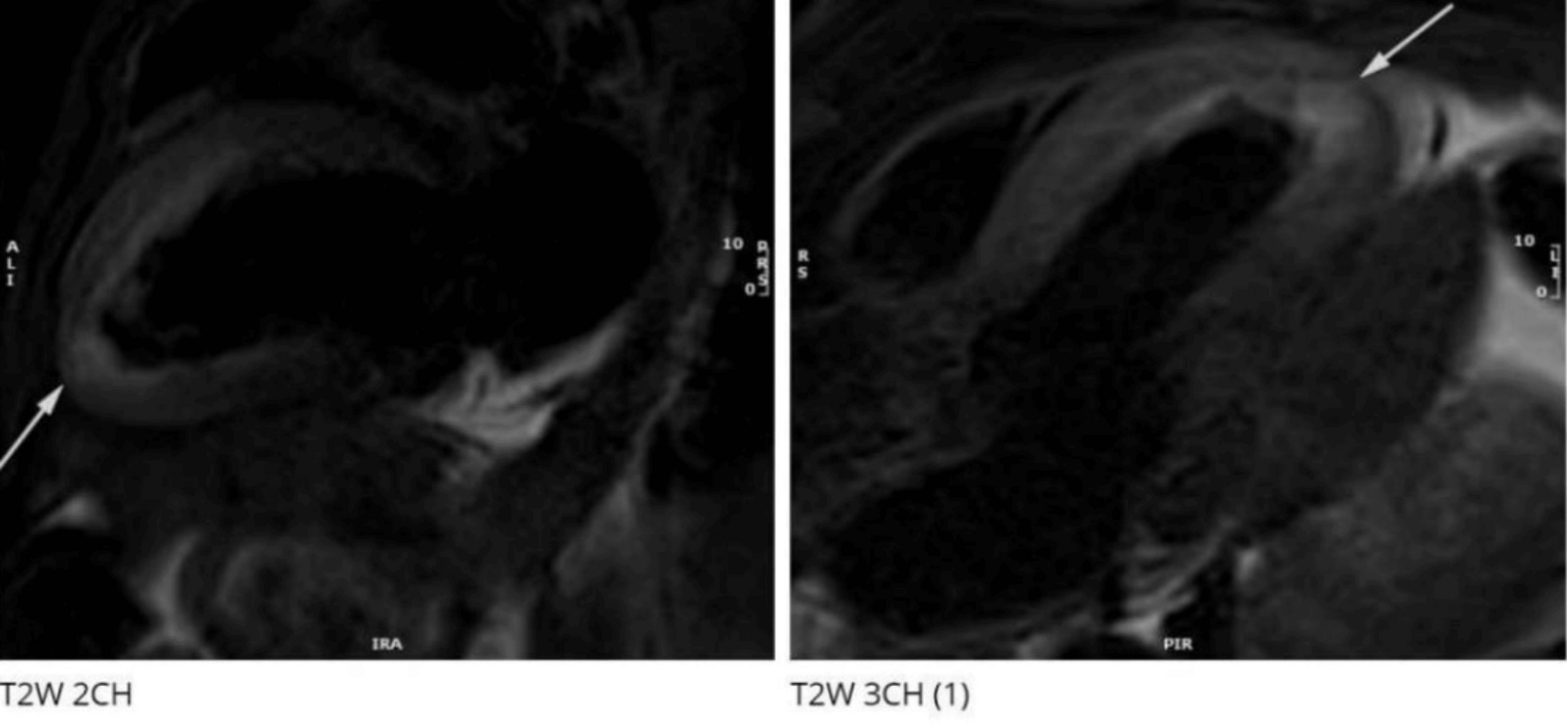
Cardiac MRI two-chamber (2CH) and three-chamber (3CH) views showing myocardial infarction in the left anterior descending artery territory. T2-weighted imaging demonstrated increased myocardial signal intensity within the mid to apical anterior wall (arrow) in the 2CH view and similar increased signal intensity in the mid to apical anteroseptum and apical cap (arrow) in the 3CH view.

Her non-contrast cardiac MRI performed a few days later showed a normal left ventricular (LV) end-diastolic volume with mildly impaired global systolic function (LVEF: 46%). Hypokinesis of the mid to apical anterior and anteroseptal segments, with severe hypokinesis of the apical cap, was observed. A small pericardial effusion was present without cardiovascular magnetic resonance (CMR) evidence of hemodynamic compromise. T2-weighted imaging demonstrated increased myocardial signal intensity within the mid to apical anterior wall, apical anteroseptum, and apical cap (Figure 4). Increased remote T2 relaxation times were also noted within the mid and apical anterior segments. In summary, findings indicated mild LV systolic dysfunction and regional wall motion abnormalities in the LAD territory with associated myocardial edema and inflammation, consistent with underlying LAD-SCAD.

During the remainder of her hospital stay, she remained chest pain-free, with no electrical or hemodynamic instability. She continued to be managed conservatively under close observation. Three weeks and five days later, she underwent an elective lower segment cesarean section under regional anesthesia and delivered a healthy baby at approximately 39 weeks of gestation.

A transthoracic echocardiogram performed on the fourth postoperative day showed a mildly dilated LV cavity with normal ejection fraction (61%), normal valvular anatomy, and normal right ventricular function.

## Discussion

SCAD is defined as the acute formation of a false lumen within the epicardial coronary arterial wall, resulting in separation of the intima from the outer layers [3]. This may occur due to either an intimal tear or disruption of the vasa vasorum, leading to intramural hemorrhage within the media without an overt intimal rupture. The resulting compression of the true lumen causes myocardial ischemia. This pathophysiological process differs from typical ACS, where plaque instability on a background of atherosclerosis is the main mechanism. Traumatic and iatrogenic causes are excluded from this definition [1].

SCAD is often associated with underlying vascular predispositions, such as fibromuscular dysplasia, and less commonly with connective tissue diseases or vasculitides. These conditions, when combined with triggering factors that increase shear stress, can initiate the dissection process. During pregnancy, elevated progesterone levels are thought to weaken the medial layer of the arterial wall. This hormonally mediated vulnerability, compounded by the physiological stresses of pregnancy, labor, and delivery, may precipitate dissection in a structurally susceptible vessel. Multiparity, assisted conception including in vitro fertilization, and preeclampsia have also been suggested as contributing factors [1].

There are no randomized controlled trials on SCAD, although ongoing prospective studies may help inform future guidelines for the diagnosis and management of P-SCAD. Until such data become available, clinicians must rely on existing recommendations and expert consensus derived from established SCAD registries. Current guidelines advocate coronary angiography as the primary diagnostic modality, with intravascular imaging such as intravascular ultrasound (IVUS) or optical coherence tomography (OCT) reserved for cases where diagnostic uncertainty persists [1]. However, these guidelines often overlook the unique considerations in antepartum patients, particularly the risks of fetal radiation exposure and contrast use.

In terms of treatment, there is strong evidence supporting conservative management, as outcomes with revascularization using PCI or coronary artery bypass grafting (CABG) have been shown to be suboptimal [3]. Revascularization is generally reserved for patients with ongoing ischemia, hemodynamic instability, or complete vessel occlusion. 

When comparing diagnostic modalities for suspected SCAD in pregnancy, both radiation and contrast exposure must be carefully weighed against their potential fetal risks. These risks should be balanced with the diagnostic benefits obtained from each modality. Theoretically, ionizing radiation is mutagenic, teratogenic, and carcinogenic [4,5], and its impact is both dose- and duration-dependent. However, the radiation doses used in current diagnostic imaging are significantly lower than the accepted upper safety limit of 5 rad for pregnant patients [6]. Similarly, while iodinated contrast agents have been associated in theory with fetal hypothyroidism, modern low-osmolar, water-soluble agents exhibit minimal placental transfer and are considered safe in pregnancy.

Invasive coronary angiogram remains the gold standard for diagnosing SCAD, particularly type I SCAD, due to its superior spatial and temporal resolution. However, the classic angiographic appearance, multiple radiolucent lumens with arterial wall staining, occurs in only about 29% of cases. The more common variants, type II and III SCAD, often require adjunctive intravascular imaging such as IVUS or OCT for definitive diagnosis [7]. Therefore, relying solely on conventional angiography may result in missing over 70% of SCAD cases [1]. Furthermore, the risk of iatrogenic dissection during a coronary angiogram in SCAD patients has been reported at approximately 2%, with an even higher risk when intravascular imaging is used [8].

Another reason for considering invasive coronary angiogram as a first-line investigation is its ability to offer therapeutic interventions. However, recent data have shown that around 80%-90% cases of SCAD are managed conservatively. Those who needed revascularization showed poor outcomes in around 50% of cases [3], which was variable in different series. Extension of dissection, no flow phenomena, and difficulty navigating the wire were common. Among those who would have successful PCI in the index cases, they can later present again with stent thrombosis due to healing of the vessel wall and resorption of the hematoma that lead to late strut malapposition [1,9].

Moreover, it can be argued that radiation and contrast exposure during invasive procedures, though variable depending on procedural complexity and the need for intervention, are typically higher than those associated with a CT coronary angiogram [10].

In contrast, CT coronary angiogram offers a non-invasive alternative that generally delivers a lower radiation dose [10], which can be further minimized through protocol optimization and field-of-view restriction, while also requiring smaller contrast volumes. Although its spatial resolution is comparatively lower [11], a CT coronary angiogram can reliably detect proximal SCAD, including left main stem involvement, without the procedural risks inherent to invasive angiography. A Canadian study demonstrated that P-SCAD most commonly involves proximal coronary arteries [12]. However, the true sensitivity and specificity of the CT coronary angiogram as a primary diagnostic tool for SCAD remain uncertain due to limited data. It is important to emphasize that in cases of ongoing ischemia or hemodynamic or electrical instability, an invasive coronary angiogram remains the first-line diagnostic approach. 

Currently, the CT coronary angiogram is primarily used for follow-up assessment of healing in previously diagnosed proximal SCAD [13]. However, due to insufficient evidence, its role as a first-line diagnostic modality in hemodynamically stable antepartum patients without atherosclerotic risk factors presenting with ACS has not been studied. To date, there are no reported cases of P-SCAD, or SCAD in general, where a CT coronary angiogram was employed as the initial diagnostic tool; most reports have relied on an upfront invasive coronary angiogram. The considerations outlined above highlight the importance of adopting individualized imaging strategies in P-SCAD, carefully balancing diagnostic accuracy with the maternal and fetal risks inherent to each modality. This case of left main stem and proximal LAD artery dissection diagnosed through CT coronary angiogram and successfully managed conservatively supports the feasibility of non-invasive diagnostic approaches in select patients. As prospective studies continue to evolve, and as more cases are managed conservatively, clearer guidance may emerge regarding the role of non-invasive imaging as a potential first-line investigation in this unique patient population.

## Conclusions

SCAD is an uncommon cause of ACS and therefore requires a high index of suspicion. In this case, the presentation of a young, pregnant woman without traditional atherosclerotic risk factors raised strong clinical suspicion for SCAD. Given the patient’s hemodynamic stability, a CT coronary angiogram was chosen to confirm the diagnosis. This represents a novel diagnostic approach in P-SCAD, as previous reports have primarily relied on invasive coronary angiography, often followed by conservative management or suboptimal outcomes following PCI or CABG. This case demonstrates that, in carefully selected and clinically stable patients with high diagnostic certainty, CT coronary angiogram can serve as a safe and effective alternative to invasive angiography, enabling diagnosis while minimizing procedural risk to both the mother and fetus.
